# Experiences of Typhus Fever
*A Paper read at the joint meeting of the Bristol Medico-Chirurgical Society and "The Galenicals," 4th May, 1942.


**Published:** 1942

**Authors:** F. Kohn

**Affiliations:** Senior Medical Officer St. Martin's Hospital, Bath.


### EXPERIENCES OF TYPHUS FEVER.*

BY

DR. F. KOHN, M.D.

*Senior Medical Officer St. Martin's Hospital, Bath.*

---

In the first month of the last war (August, 1914), a young man not yet qualified, I was acting as intern at a hospital in Chomutov. I admitted a middle-aged man apparently unconscious, but very excited, with a flushed face, and injected conjunctivæ. The patient's temperature was very high, but there were no physical signs in the chest or abdomen to account for it. The senior physician of the hospital came and looked at the man and diagnosed the condition at a glance. "A very interesting case, typhus exanthematicus." The patient became more deeply unconscious, and in three days was dead.

At that time very little was known about typhus; a few people talked about insect carriers, possibly the flea or louse. After the first patient was admitted, nothing happened for a fortnight. Then two nurses and a doctor in the hospital were taken ill with the same symptoms. On the next day a fair number of people living in the same place as the original patient came into the hospital presenting the same symptoms.

This sequence is followed in every outbreak of typhus in a new place. A single case followed at an interval of a fortnight with a group of new cases: and presently, unless drastic measures are taken, there will be a general spread to the whole community.

In England you are very fortunate, you have hardly ever seen a case. If you have seen only a few cases you can never miss typhus.

The history is one of sudden onset with headache, great weakness, "feeling rotten." The temperature soon rises very high and the patient becomes very prostrate, weak and miserable. Soon he becomes confused, in a haze as it were. Sometimes the appearances are like meningitis, sometimes like pneumonia. On the third or fourth day the exanthem shows, chiefly in the axillary folds with a

* A Paper read at the joint meeting of the Bristol Medico-Chirurgical Society and "The Galenicals," 4th May, 1942.

few spots on the abdomen. If the case is mild the spots are few—they look more or less livid and may be distinguished from those of typhoid by the fact that typhus spots cannot be made to disappear by pressure, but those of typhoid vanish. If the spots are few they might be taken for flea bites, but the typhus spot never has in its centre a bleeding point as does a flea or louse bite. If the case is serious the rash quickly becomes haemorrhagic and spreads all over the back and thighs. I have never seen spots on the face: in addition to the exanthem the patient's face is not only flushed but definitely swollen like oedema, and there are signs of conjunctivitis, or conjunctival congestion. The patient's behaviour is characteristic. He is unconscious, yet at the same time agitated, talking and always doing something : eventually he may become stuporous and die.

The mortality is terrific. In patients over 60 years of age more than 80 per cent. die, in middle age the prognosis is better, but it is best in children.

At the beginning of 1915 Prowazek first announced his discovery that the louse was the carrier of typhus, and Prowazek corpuscles were identified as the agent of typhus. The proof lay in the experiment of allowing lice in an ivory case to feed on an infected person : these corpuscles, previously absent, were invariably found in the bodies of the lice so fed.

Now began the fight against the louse. People were examined compulsorily. If lice were found on them (or more correctly in their clothes, for they are rarely seen on the skin itself) all the hair on their bodies, as well as their beards and heads were shaved. Their clothing, bedding and houses were thoroughly deloused. This stopped the epidemic.

There were many sporadic cases, but the epidemics in prison camps were dreadful. For example, of 30,000 prisoners from Serbia 10,000 died from typhus alone.

In 1915 I joined the Army and went to Poland. My colleagues laughed at my apprehensions. Typhus, they said, was always present there. It was true. Almost every house had someone ill with typhus, and it did not seem to matter so long as it was only a family disease. The type seemed milder. But soon it spread amongst the troops and then it was a much more serious disease.

I started the same drastic measures and saved the troops from becoming infested and infected. The war in Poland was very mobile, the Army retreated and advanced many times over the same ground which made disinfestation very difficult.

Disinfection was carried out with steam at a pressure of 2½ atmospheres.

The disinfecting chamber was placed close to the baths. Patients showed lice of all types and it was questioned which type was the carrier. It is now thought that only the body louse conveys typhus, but some authorities believe the head louse is also capable of doing so. Nothing is known about the pubic louse in relation to typhus. If a thickly populated area becomes infested with lice and only a few cases of typhus are introduced, an epidemic will arise which will be difficult to control.

Since no treatment is of any effect the only possible way of controlling typhus is to fight against the carrier, that is, against the louse.

No anxiety need be felt about typhus if delousing can be done. Last year I saw many workmen from a neighbouring district heavily infested with lice, but local arrangements for delousing have been made, with the result that within the last two months, I have not seen a single case from those works with lice.

The complications of typhus are few. Broncho-pneumonia, parotitis and orchitis are fairly common. I have seen two cases in which gangrene of the lip occurred, possibly from arterial embolism, also two cases of amaurosis; the Matron of a hospital and a soldier went quite blind.

The typhus endemic in Galicia is much milder than in Bohemia. The fever subsides after 14 days, but the muscular prostration which is so severe may last for months. I have never seen a second attack.

The diagnosis has been made easier by the Weil-Felix reaction, but the experienced doctor does not have to wait for the Weil-Felix result before making a diagnosis. Splenic enlargement is slight or absent in typhus. The excretion of chlorides in the urine is an aid to prognosis. If the output of chlorides is good, there is a good prospect of recovery.

### DISCUSSION.

In reply to a question by Professor Perry, Dr. Kohn said that the virulence of typhus was increased where an epidemic broke out amongst an ill-nourished population. He reminded the meeting that this was implied in the old name "famine fever."

Dr. Frank Bodman asked if the clinical picture of typhus in a child differed from that in an adult. Dr. Kohn replied that the clinical picture was the same but never so severe in a child, and the rash was never hæmorrhagic.

Dr. Orr Ewing asked if there was a characteristic odour. Dr. Kohn said he thought that the odour was more characteristic of the patient's environment than of the disease.

Mr. Wilfrid Adams inquired about the post-mortem appearances. Dr. Kohn said that opportunities for autopsies in the Army or in the epidemics he had seen were very few. There was nothing very typical. But it was most striking that a louse never remained on a dead body. He mentioned a characteristic lesion which could be demonstrated during life as well as after death. In the centre of an excised papule definite periarteritis can be seen. In Prague this was used as a method of diagnosis before the Weil-Felix reaction was introduced.

Replying to Mr. Angell James, Dr. Kohn said that Professor Hoke (who translated Osler's *Medicine* into Czech) ordered protective clothing. It was mainly rubber mackintosh, and so nasty to work in that it was given up. However, if the patient is properly deloused, and the ward is kept free from lice, there is no danger to the ward attendants.

Dr. Rayner asked if one attack protects. Dr. Kohn thought that this was the case.

